# Radiomic-based prognostic score for survival risk-stratification in pediatric medulloblastoma tumors: A multi-institutional study

**DOI:** 10.1093/noajnl/vdaf107

**Published:** 2025-06-05

**Authors:** Marwa Ismail, Hyemin Um, Gustavo Pineda, Ralph Salloum, Fauzia Hollnagel, Raheel Ahmed, Peter de Blank, Pallavi Tiwari

**Affiliations:** Department of Radiology, University of Wisconsin-Madison, Madison, Wisconsin; Department of Radiology, University of Wisconsin-Madison, Madison, Wisconsin; Department of Radiology, University of Wisconsin-Madison, Madison, Wisconsin; Dana-Farber/Boston Children’s Cancer and Blood Disorders Center, Harvard Medical School, Boston, Massachusetts; Department of Medicine, University of Wisconsin-Madison, Madison, Wisconsin; Department of Neurological Surgery, University of Wisconsin-Madison, Madison, Wisconsin; Department of Pediatrics, Cincinnati Children’s Hospital Medical Center, Cincinnati, Ohio; Departments of Medical Physics and Biomedical Engineering, University of Wisconsin-Madison, Madison, Wisconsin

**Keywords:** heterogeneity, medulloblastoma, penalized cox proportional hazards models, radiomics, risk

## Abstract

**Background:**

Medulloblastoma (MB) is the most common malignant brain tumor in children and is known for substantial heterogeneity. MB is classified into low/average- and high-risk; however, improving risk-stratification remains one of the biggest challenges in MB. Enriching risk-stratification offers potential treatment intensification for high-risk MB while decreasing treatment sequalae in low-risk MB through treatment de-escalation. This work presents a new radiomics-based medulloblastoma prognostic (RaMP) score that quantifies the tumor heterogeneity by analyzing routine clinical imaging. The hypothesis is that radiomic (computational) features can analyze the complex behavior and heterogeneity of MB tumors, allowing for segregating high-risk tumors from the ones with low risk.

**Methods:**

One hundred and nineteen MRI scans for MB patients were collected from 3 institutions (Site 1: *n* = 42, Site 2: *n* = 47, Site 3: *n* = 30). Following segmentation of the tumor habitat (edema, enhancing lesion, and non-enhancing tumor + cystic core) and pre-processing, features capturing (1) structural deformations from the normal regions around the tumor, (2) textural attributes, and (3) morphological changes, were extracted. RaMP was created by combining the features then feeding them to Cox proportional hazards models, categorizing the subjects into low and high risks.

**Results:**

RaMP outperformed clinical standard-of-care assessment when employed for risk-stratification. For instance, when using Sites 1 and 3 for training and Site 2 for testing, significant differences were observed across edema and the tumor habitat with *P* = .02, .013, and *C*-indices of 0.7, 0.63, respectively, between the risk groups.

**Conclusions:**

Our work shows the promise of radiomic analysis in risk-stratification and predicting outcomes in pediatric MB.

**Key Points**

- RaMP was optimized, evaluated on medulloblastoma data from 3 different sites (*n* = 119).
- The 3 sites were used interchangeably for training and testing to evaluate RaMP.
- RaMP outperformed models using individual radiomic features for risk-stratification.

**Importance of the Study**

Medulloblastoma is a malignant brain tumor in children with high treatment-related morbidities. Treatment strategy primarily depends on the patient’s risk level (low/average, high). The current risk assessment relies on molecular stratification into 4 subgroups (WNT, SHH, Group 3, Group 4). Research on risk-stratification, however, is still evolving due to wide disparities in prognosis among patients in the same subgroups. Consequently, there is a detrimental impact on the quality of life of survivors due to the development of late treatment sequalae. This work presents a radiomic-based prognostic score to enrich risk-stratification in medulloblastoma. Our approach seeks to characterize the tumor and capture its heterogeneity that is key to aggressiveness and poor outcomes via leveraging radiomic features extracted from the tumor sub-compartments on clinical MRI scans. Our score could provide complementary information to identify high-risk cases who may benefit from intensified therapy from those at low/average-risk who merit for tailored treatments.

Medulloblastoma (MB) is the most frequent malignant brain tumor in children, accounting to over 20% of all pediatric intracranial tumors. Its overall survival remains inadequate with a 5-year survival rate of around 70% to 75%.^1^ Relapse in MB is nearly always fatal, making the proper selection of upfront chemotherapy and/or craniospinal irradiation based on risk-stratification critical.^2^ Currently, children over 3 years of age who have minimal residual disease after tumor resection and no evidence of central nervous system dissemination at diagnosis are considered “average-risk” and have survival rates of 80% to 85%.^3^ While the survival rates are high for average-risk patients, when overtreated, those survival rates come at the expense of long-term toxicities and cognitive sequelae from therapy. Specifically, around 57% of MB survivors develop at least one severe, life-threatening, or fatal chronic health condition due to therapy.^3,4^ Such late effects include, but are not limited to, hearing loss,^4–6^ vision problems,^4,5^ seizure disorders,^5^ development of secondary cancers,^4,6,7^ and ovarian failure^8^.

Recent clinical trials have been designed to decrease the toxicity of treatment in children diagnosed with MB, incorporating the current risk-stratification approaches.^3,9^ These approaches include (1) Chang’s classification^7,10^ which uses clinical parameters to stratify patients into average-risk and high-risk, and more recently, (2) molecular classification^11–15^ that categorizes patients into 4 distinct subgroups (Wingless [WNT], Sonic Hedgehog [SHH], Group 3, and Group 4). Chang’s classification relies on clinicopathological criteria, such as age at diagnosis, presence of metastasis, and extent of resection along with the histological variants/subtypes of the tumor. Molecular subgroup classification is the most recent risk assessment strategy and is currently guiding MB clinical trials and showing potential for targeted therapies. Though the 4 subgroups are proven to have distinct transcriptional profiles, copy-number aberrations, somatic mutations, and clinical outcomes,^14^ there exist wide disparities in outcomes among patients within the same subgroup. This has led to further exploration of stratification of each subgroup into subtypes for the sake of personalized therapies,^14,16^ with continued emergence of subtypes within each subgroup in different studies without a consensus on the number of subtypes (7 to 12 subtypes reported within the 4 subgroups). In addition, molecular tests are not always available outside major cancer centers, and with turnaround times of weeks that may lead to patient anxiety and delayed treatment.^17^ Consequently, there is a critical need for complementary, non-invasive prognostic tools to reliably risk-stratify patients to improve treatment outcomes in MB patients.

Radiomics (extraction of quantitative features from imaging) provides a surrogate mechanism to non-invasively capture the subvisual cues of intra-tumoral heterogeneity on routine MR imaging (MRI) and has demonstrated prognostic significance across different types of tumors^18–22^ (including pediatric brain tumors^23–34^). Traditional radiomic measurements have shown success in predicting MB subtypes and overall outcomes in recent studies.^24–34^ While morphology is important for tumor characterization, presence of mass effect has also been shown to be associated with aggressive tumor behavior and poor prognosis in MB tumors,^35^ but still severely understudied in the context of pediatric brain tumors (including MB).

In this work, we present a radiomics-based MB prognostic (RaMP) score to predict MB outcomes by leveraging radiomic features that measure several attributes that capture the intra- and peri-tumoral heterogeneity as well as quantify the mass effect in MB, from the tumor sub-compartments, the enhancing lesion, the non-enhancing tumor + cystic core, and peri-tumoral edema (together all 3 are called “tumor habitat”), on clinical MRI scans (T1w, T2w, FLAIR). We hypothesize that, (1) the deformation heterogeneity attributes on MRI may provide prognostic insights into MB aggressiveness level, and (2) coupling the deformation heterogeneity features with other texture and morphological features may improve patient prognosis in MB tumors. Uniquely, our analysis is conducted in an age-specific manner, to account for the anatomy of the developing brains across the different life stages. We validate the RaMP score on multi-institutional data as well as conduct comparative analyses to assess its prognostic efficacy against current survival risk assessment approaches, including clinicopathological and molecular criteria.

## Materials and Methods

### Methods Overview

**Figure 1.**
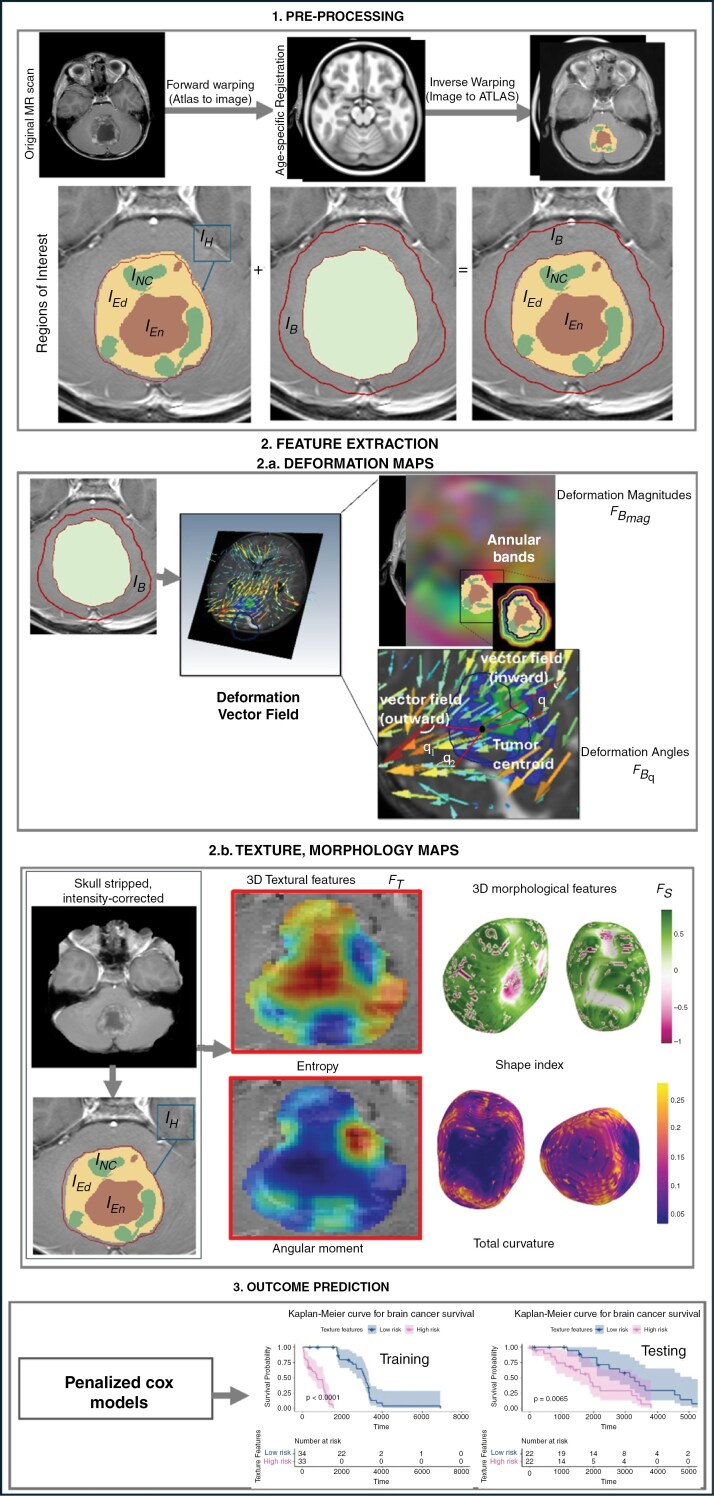
Pipeline of RaMP score and its application on pediatric MB risk-stratification.

Figure 1 illustrates the pipeline of our framework. Briefly, following pre-processing, which includes registration of the scans to age-appropriate atlases and expert segmentations of the tumor sub-compartments (enhancing lesion, peri-tumoral edema, and non-enhancing tumor + cystic core), textural and shape features are extracted from the “tumor habitat” (a union of the 3 sub-compartments), as well as individually from each sub-compartment, while deformation heterogeneity measurements are extracted from the regions outside the tumor habitat. This is followed by incorporating these features into penalized Cox proportional hazards models to construct the RaMP score for MB risk-stratification and compare it to overall survival. Table 1 includes a list of notations and acronyms used in this paper.

**Table 1.** List of Notations and Acronyms in this Paper.

Notation	Description	Notation	Description
$I_{En}$	Enhancing lesion region	$S_{v}$	Test set
$I_{Ed}$	Edema region	$F_{\;}$	Feature set
$I_{NC}$	Necrotic + cystic core regions	${F_{Mag}}^{B}$	Deformation magnitude descriptor
$I_{H}$	Tumor habitat	${F_{\theta }}^{B}$	Deformation orientation descriptor
$I_{B}$	Normal parenchyma sub-volumes	$F^{T}$	Texture descriptor
$S_{t}$	Training set	$F^{S}$	Shape descriptor
${F_{En}}^{S}$	Shape features for the enhancing lesion region	${F_{H}}^{S}$	Shape features for the tumor habitat
${F_{Ed}}^{S}$	Shape features for the edema region	${F_{En}}^{T}$	Texture features for the enhancing lesion region
${F_{NC}}^{S}$	Shape features for the necrotic + cystic core regions	${F_{Ed}}^{T}$	Texture features for the edema region
${F_{H}}^{T}$	Texture features for the tumor habitat	${F_{NC}}^{T}$	Texture features for the necrotic + cystic core regions
$N_{t\;}$	Number of subjects in a training set	$N_{v}$	Number of subjects in a test set
$F_{I}$	Integrated Feature Set	${S_{t}}^{S\text{1}}$	Training set using Site 1 data
${S_{t}}^{S\text{2}}$	Training set using Site 2 data	${S_{t}}^{S\text{3}}$	Training set using Site 3 data
${S_{t}}^{S\text{1,}2}$	Training set using Sites 1,2 data	${S_{t}}^{S\text{1,}3}$	Training set using Sites 1,3 data
${S_{t}}^{S\text{2,}3}$	Training set using Sites 2,3 data	${S_{v}}^{S\text{1}}$	Test set using Site 1 data
${S_{v}}^{S\text{2}}$	Test set using Site 2 data	${S_{v}}^{S\text{3}}$	Test set using Site 3 data

Acronym	Description	Acronym	Description
CBTN	Children’s Brain Tumor Network	CI	Confidence Interval
HR	Hazard Ratio	KM	Kaplan–Meier
*C*-index	Concordance Index		

### Notation

We define an image scene I as I = (C,f), where I is a spatial grid of voxels c ∈C, in a 3-dimensional space, $R^{3}$. Each voxel, c ∈C, is associated with an intensity value f(c). We also define $I_{En},\;I_{Ed},\;I_{NC},\;I_{H}\;$ corresponding to the enhancing tumor sub-compartment, the peri-tumoral edema sub-compartment, the necrotic + cystic core sub-compartment, and the tumor habitat that comprises the 3 tumor sub-compartments, respectively. $I_{B}$ represents the surrounding normal parenchymal sub-volumes within every I, such that $\left[I_{En},\;I_{Ed},\;I_{NC},\;I_{H},\;I_{B}\;\right]\;\subset I$. We further divide the sub-volume $I_{B}$ into uniformly sized annular sub-volumes $I_{B_{a}}$, where a is the number of uniformly sized annular bands representing the healthy regions immediate to the tumor, such that a ∈ {1,…,m}, and m is a user-defined proximity parameter that is dependent on the distance from the tumor margin. We extract each feature set F from each training $S_{t}$ and test $S_{v}\;$ set, across the different cohorts employed in this study.

### Computation of Radiomics features

Below, we provide details on each feature family and how they are integrated to construct the RaMP score.

#### Deformation heterogeneity features from the normal parenchyma

($I_{B}$)—To compute the structural deformations occurring in $I_{B}$, all the scans of the diseased cohorts were aligned to age-specific healthy atlases^36^ using a diffeomorphic scheme executed using ANTs (Advanced Normalization Tools) toolbox.^37^ Our age-specific registration scheme guarantees that the anatomical changes throughout the different developing phases of life (2 to 21 years of age) are accounted for. The tumor masks were removed from the subjects during the registration process to recover only the spatial intensity differences due to structural deformations when compared to the atlases. Next, the subject brains were warped to the atlas space, using the inverse mapping of the registration, to compute subtle per-voxel structural deformations. Next, to quantify the localized deformation changes around the tumor’s infiltrating edge, the region outside the infiltrating T2/FLAIR hyperintensities were divided into equidistant annular bands from the tumor edge to the skull boundary, $I_{B_{a}}$. Our feature sets, consisting of both magnitude and phase of the deformation vectors, were then obtained.

Firstly, we extract per-voxel deformation magnitudes within these bands by calculating the Euclidean norm of the scalar values of the deformation fields. Then, 5 statistics were obtained (ie mean, median, standard deviation, skewness, and kurtosis of the deformation magnitudes) to acquire a 5 × 1 deformation magnitude vector for each band, $F{{}_{B}^{Mag}}_{\;}$.

Secondly, we quantify the per-voxel deformation orientations by computing the deformation angle θ between each deformation vector x and the vector that connects each voxel to the tumor’s centroid y, using the formula $\theta =\arccos\left(\frac{x.y}{\left|x\right|\left|y\right|}\right)$. The deformation vector fields that have an outward direction of tissue displacements, ie positive-valued angles with respect to the tumor’s borders will likely reflect the shift that the tumor causes in the healthy regions due to mass effect and hence may be linked to more aggressive tumors (eg $\theta_{1},\;\theta_{2\;}$ in Figure 1). Conversely, the less aggressive tumors will likely exhibit an inward orientation with negative-valued angles with regards to the tumor borders (eg $\theta_{3}\;$Figure 1). After computing angles of all the deformation fields in every annular band, angle values were quantized using a fixed width binning technique using 6 intervals ([0° to 29°], [30° to 59°], [60° to 89°], [90° to 119°], [120° to 149°], [150° to 179°]), creating a 6 × 1 orientation vector for each band, $F_{B}^{\theta }$. Finally, the deformation magnitude vector as well as the deformation orientation vector within each of the created annular bands are aggregated into a 11 × 1 vector to construct $F_{B_{\;}}$ constituting [$F{{}_{B}^{Mag}}_{\;},\;F_{B}^{\theta }$]. For the deformation features, $F{{}_{\;}^{B}}_{\;},$, we computed $F{{{}_{Mag}^{B}}_{\;}}_{\;}$ and $F{{}_{\theta }^{B}}_{\;}$ for a   ∈{1, 2, 3, …,12} annular concentric bands that are 5 mm apart (this distance a recommended clinical target volume margin for brain tumors^38^). Computing the deformation feature attributes for each band resulted in a 192 × 1 vector that comprises: (1) a 60 × 1 vector ($F{{}_{Mag}^{B}}_{\;}$) with 5 statistics (mean, median, standard deviation, skewness, and kurtosis) per band, (2) a 11 × 1 vector ($F{{}_{\theta }^{B}}_{\;}$) with the same 5 statistics for the angles per band, and (3) a 72 × 1 vector (nb) that included the number of bins for each of the 6 quantized angle intervals, per band. This ultimately resulted in the formation of $F{{}_{\;}^{B}}_{\;}$, a 192 × 1 vector that is extracted for each tumor region $I_{En},\;I_{Ed},\;I_{NC},\;I_{H}$, for each of the 3 MRI protocols.

#### Textural features from the tumor sub-compartments

($I_{En},\;I_{Ed},\;I_{NC}$) and the tumor habitat $I_{H}$—A total of 214 texture features including gradient, Haralick, Laws, Gabor, and COLLAGE features,^39,40^ are computed for every $c\;\in \;\left\{C_{En},\;C_{Ed},\;C_{NC},C_{H}\right\}$. We then utilized these per-voxel measurements to compute first-order statistics (mean, median, standard deviation, skewness, and kurtosis) per feature for every tumor region. This resulted in the feature descriptors $F_{En}^{T},\;F_{Ed}^{T},\;F_{NC}^{T},\;F_{H}^{T}$, representing the texture descriptor, $\;F_{\;}^{T},\;$ for each tumor sub-compartment. For the textural features, $F{{}_{En}^{T}}_{\;},\;F{{}_{Ed}^{T}}_{\;},\;F{{}_{NC}^{T}}_{\;},\;F{{}_{H}^{T}}_{\;}$, a total of 5 statistics were calculated for each of the 214 extracted features, resulting in a 1070 × 1 vector, per each tumor region $I_{En},\;I_{Ed},\;I_{NC},\;I_{H}$, for each of the 3 MRI protocols.

#### Shape features from the tumor sub-compartments

($I_{En},\;I_{Ed},\;I_{N+C}$) and the tumor habitat $I_{H}$—A total of 18 morphological features are extracted from the different tumor sub-compartments. Specifically, 4 local features that capture surface-based irregularities (Curvedness, Sharpness, Shape Index, and Total Curvature) were computed from $I_{En},\;I_{Ed},\;I_{NC},\;I_{H}$, individually.^41^ In addition, 14 morphological features capturing the global contour characteristics were computed from $I_{En},\;I_{Ed},\;I_{NC},\;I_{H}$, individually. The features are volume, major axis length, minor axis length, eccentricity, elongation, orientation, perimeter, roundness, equivalent spherical radius, equivalent spherical diameter, flatness, elongation shape factor, compactness, and integrated intensity.^41^ These attributes resulted in the feature descriptors $F{{}_{En}^{S}}_{\;},\;F{{}_{Ed}^{S}}_{\;},\;F{{}_{NC}^{S}}_{\;},\;F{{}_{H}^{S}}_{\;}$, representing the shape descriptor, $F_{\;}^{S},\;$ for each tumor sub-compartment and the tumor habitat. For the shape features $F{{}_{En}^{S}}_{\;}\;F{{}_{Ed}^{S}}_{\;},\;F{{}_{NC}^{S}}_{\;},\;F{{}_{H}^{S}}_{\;}$, the 5 statistics were calculated for each of the 4 extracted surface-based features, and then concatenated with the 14 global features, resulting in a 34 × 1 vector, per each tumor region $I_{En},\;I_{Ed},\;I_{NC},\;I_{H}$.

All features were subsequently aggregated, resulting in an integrated feature set, $F_{I}$, with a total of 192×3+1070×3+34=3820 features, for each of $I_{En},\;I_{Ed},\;I_{NC},\;I_{H}$, for cases where all 3 MRI modalities were available. For the cases that only had Gd-T1w sequences available, $F_{I}$ included 192+1070+34=1296 features.

### Experimental Design

#### Study population and confirmation for disease presence

A total of 119 MB studies were retrospectively collected and ranged between 2 and 18 years in age. The MRI scans of the MB cases were obtained from 3 different institutions: Site 1 (*n* = 42), Site 2 (*n* = 47), and Site 3 (*n* = 30). The scans were performed from the year 2000 up to the date of investigational review board (IRB) approved data (May 16, 2019). Retrospective review of clinical data was reviewed by the IRB of participating sites and a waiver of informed consent was obtained. Subject age, sex, tumor characteristics (including Chang classification and molecular subgroup), as well as vital status and time from diagnosis at last follow up were abstracted from the clinical record. The studies were acquired using 1.5 and 3 T MRI Philips (Ingenia, Achieva) and Siemens scanners. The inclusion criteria used for our datasets were: (1) availability of Gd-T1w axial view MRI scans; (2) patients with only MB tumors; (3) acceptable diagnostic quality of the MRI scans, as identified by the collaborating radiologists; and (4) availability of overall survival information, Chang’s classification, and molecular subgroup information. While Gd-T1w scans were available for all 119 studies, all 3 MRI sequences Gd-T1w, T2w, and FLAIR were available for a subset of the studies (*n* = 83). including 35 cases from Site 1, 18 cases from Site 2, and 30 cases from Site 3. Table 2 shows further details pertaining to the demographics and the scanner information of study participants. To ensure rigorous validation of our descriptor, we utilized the data in 3 different combinations, for the experiments conducted in this work, where we used 2 of the 3 independent datasets for training ($S_{t}$) and used the third one independently for testing ($S_{v_{\;}}$), at a time.

**Table 2.** Patient Demographics and MRI Acquisition Information Across Our Multi-institutional Data.

		Site		
		Site 1	Site 2	Site 3
*N*		42	47	30
Age, mean (SD)		7.85 (4.5)	6.6 (4.3)	9.1 (4.1)
Sex	Male	33 (78.5%)	28 (59.5%)	21 (70%)
	Female	9 (21.5%)	19 (40.5%)	9 (30%)
**Image acquisition parameters**				
Scan type		T1-FFE axial post-contrast	T1-FFE axial post-contrast	T1-FFE axial post-contrast
MR acquisition type		2D	2D	2D
Scanning sequence		Gradient-recalled	Gradient-recalled	Spin-echo
Sequence variant		Steady-state	Steady-state	Segmented *k*-space/Spoiled/Oversampling phase
Pixel spacing (mm)		0.46 to 1	0.46 to 1	2
Slice thickness (mm)		Mean = 5.4	Mean = 5.4	Mean = 5.4

#### Pre-processing

Ground truth annotations for all MB cases were generated via consensus across 2 experienced board-certified neuro-radiologists (Expert 1 with 9 years of experience and Expert 2 with 8 years of radiology experience) using 3D Slicer.^42^$I_{En}$ was defined as the hyperintense region appearing on Gd-T1w image while $I_{Ed}$ was defined to be bright on T2w and FLAIR scans. $I_{NC}$ was identified as gray/dark on Gd-T1w and FLAIR scans, with the cystic core sub-compartment to be hyperintense on T2w scans. Finally, $I_{H}\;$ was defined as the union of the 3 tumor sub-compartments $I_{En},\;I_{Ed},\;I_{NC}$.

Following segmentations, we performed a registration process for the pediatric MB scans, where we employed age-specific atlases to account for the anatomical differences across the different age groups due to brain development in pediatric patients. A total of 4 age-specific atlases (0 to 2, 2 to 5, 5 to 10, 10 to 18 years) were used.^36^ We first registered the Gd-T1w images to the age-specific atlases using a diffeomorphic registration approach and then registered the corresponding T2w and FLAIR scans to the Gd-T1w atlas-registered scan, to align all MRI protocols to the same reference space. Skull stripping was simultaneously conducted. Finally, bias correction and intensity matching approach^43^ were applied to all the scans, where inhomogeneity correction was applied, followed by a *z*-score normalization step to standardize the intensity values in all the MRI scans, bringing them all at the same intensity range. These last steps were critical in our analysis, to account for the variations across sites’ scanners and the different tesla strengths the studies were acquired with (1.5 T for Sites 1 and 3, and 3 T for Site 2 cases).

#### Statistical analysis for survival risk assessment

Prior to survival risk assessment, Spearman’s correlation was applied to remove redundant features within each family and only keep the highly uncorrelated features. Then, all the feature attributes were employed within penalized Cox proportional hazards models, with the following penalties: (1) Least Absolute Shrinkage and Selection Operator (LASSO)-Cox (L1-regularization, penalty term, α=1), (2) ridge-Cox (L2 regularization, α=0), and (3) elastic net (combining the penalty terms of both LASSO and ridge regression, 0< α<1) to conduct survival risk assessment.^44^

Specifically, those models, with their different regularization approaches, are utilized to handle high-dimensional data and conduct feature selection on our integrated feature set, $F_{I}$, then create a continuous survival risk score for the MB cases, RaMP, defined as: $=\;\sum_{z=1}^{Ft}co_{z}F_{\gamma }^{z}$, where Ft is the number of features selected by the regression model, $F_{\gamma }^{z}$ is the zth feature for $\;\gamma =\left[B,\;En^{T},\;Ed^{T},\;NC^{T},\;H^{T},\;En^{S},\;Ed^{S},\;NC^{S},\;H^{S}\right]$, and $co_{z}$ is the respective beta coefficient in the regression model. A RaMP score was obtained per patient based on the threshold value provided by the fitted regression model, to stratify patients into high-risk and average-risk groups on $S_{t}$. Log-rank test along with Kaplan–Meier (KM) survival analysis were then performed. Additionally, performance metrics were computed to assess the efficacy of our survival prognostication models, such as hazard ratios (HR), risk of experiencing the event of interest at a time point,^45^ 95% Confidence Interval (CI), level of uncertainty about the point estimates,^46^ and Concordance index (*C*-index), a measure of the probability of concordance between the predicted and the observed survival.^47^ All the computations were conducted using RStudio (V.4.3.1). Finally, the top features that resulted in significance between the risk groups on $S_{t}$ were used on $S_{v}$ to calculate RaMP for every patient, followed by the log-rank test to obtain the level of significance between the 2 identified risk groups.

In addition, we assessed whether there are any statistical associations between RaMP and the clinical approaches used for risk-stratification, ie Chang’s classification and molecular subgroup classification. Specifically, we applied McNemar test^48^ for correlated proportions, by building the 2 × 2 contingency tables that show the number of high-risk and low-risk patients identified by RaMP and Chang’s classification. Further, we applied Chi-square test of independence^49^ to determine if there are statistical associations between RaMP and molecular subgroup classification.

### Comparative Strategies

We performed multiple experiments to compare the efficacy of RaMP to the risk scores obtained by other combinations of radiomic features as well as the current clinical and molecular stratification approaches. In addition, we examined the efficacy of complementing RaMP with the current risk-stratification approaches. Specifically, experiments with the following feature attributes were conducted for survival risk assessment: (1) Chang’s stratification, (2) molecular stratification, (3) $F_{\;}^{T}$ alone, (4) $F_{\;}^{S}$ alone, and (5) $F_{\;}^{B}$ alone. For the comparisons to the molecular stratification scheme, since our data was curated when the stratification into 4 distinct subgroups only (WNT, SHH, Group 3, Group 4) was available, our comparative experiments were based on that molecular scheme with the subgroup classification,^17^ not the subtype classification. Table 3 lists all the experiments conducted across the different training and test sets, with the number of participants, the feature families employed, and the MRI protocols involved in each experiment.

**Table 3.** Experiments conducted in this work with regard to the feature family, the training datasets, and the test datasets.

Feature family	Extracted features (per protocol per tumor region)	Training sets schemes and number of participants					
		$S_{t}^{S1,2}$ , $S_{v}^{S3}$		$S_{t}^{S1,3}$ , $S_{v}^{S2}$		$S_{t}^{S2,3}$ , $S_{v}^{S1}$	
		Gd-T1w	3 protocols	Gd-T1w	3 protocols	Gd-T1w	3 protocols
Deformation ($F_{B}$)	**192**: 60 for magnitudes, 60 for angles, 72 for angle intervals bins	$N_{tot}=119$ $N_{t\;}=89$ $N_{v}=30$	$N_{tot}=83$ $N_{t\;}=53$ $N_{v}=30$	$N_{tot}=119$ $N_{t\;}=72$ $N_{v}=47$	$N_{tot}=83$ $N_{v}=18$	$N_{tot}=119$ $N_{t\;}=77$ $N_{v}=42$	$N_{tot}=83$ $N_{t\;}=48$ $N_{v}=35$
Texture ($F_{T}$)	**1070**, including Collage, laws, Gabor, Haralick, gradient	$N_{tot}=119$ $N_{t\;}=89$ $N_{v}=30$	$N_{tot}=83$ $N_{t\;}=53$ $N_{v}=30$	$N_{tot}=119$ $N_{t\;}=72$ $N_{v}=47$	$N_{tot}=83$ $N_{t\;}=65$ $N_{v}=18$	$N_{tot}=119$ $N_{t\;}=77$ $N_{v}=42$	$N_{tot}=83$ $N_{t\;}=48$ $N_{v}=35$
Shape ($F_{S}$)	**34**, including 20 surface-based and 14 global features (for all protocols together)	$N_{tot}=119$ $N_{t\;}=89$ $N_{v}=30$	$N_{tot}=83$ $N_{t\;}=53$ $N_{v}=30$	$N_{tot}=119$ $N_{t\;}=72$ $N_{v}=47$	$N_{tot}=83$ $N_{t\;}=65$ $N_{v}=18$	$N_{tot}=119$ $N_{t\;}=77$ $N_{v}=42$	$N_{tot}=83$ $N_{t\;}=48$ $N_{v}=35$
RaMP ($F_{I}$)	($F_{B},\;F_{T},\;F_{s}$) **1296** for 1 protocol, **3820** for 3.	$N_{tot}=119$ $N_{t\;}=89$ $N_{v}=30$	$N_{tot}=83$ $N_{t\;}=53$ $N_{v}=30$	$N_{tot}=119$ $N_{t\;}=72$ $N_{v}=47$	$N_{tot}=83$ $N_{t\;}=65$ $N_{v}=18$	$N_{tot}=119$ $N_{t\;}=77$ $N_{v}=42$	$N_{tot}=83$ $N_{t\;}=48$ $N_{v}=35$

## Results

### Survival Risk Assessment Using RaMP

**Figure 2.**
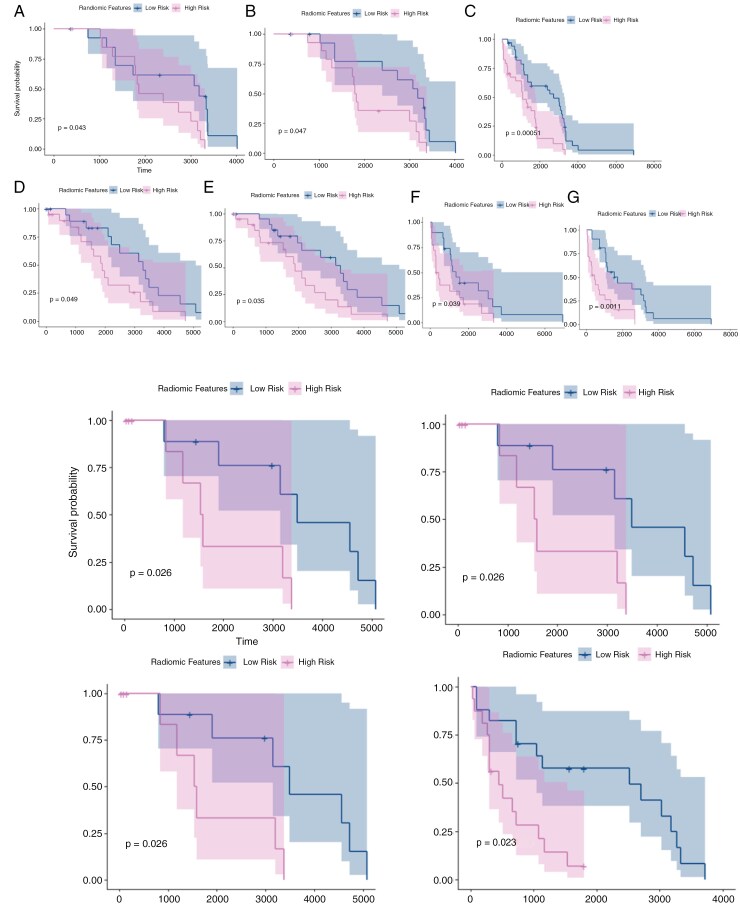
**A**) Kaplan–Meier curves that show the risk-stratification results on the test sets when employing all 3 feature families together (Shape-, texture-, deformation-based) on the 3 data combinations. These experiments were performed on subjects that only had the Gd-T1w scans available. **B**) Kaplan–Meier curves that show the risk-stratification results on the test sets when employing all 3 feature families together (Shape-, texture-, deformation-based) on the 3 data combinations. These experiments were performed on subjects that had all the 3 modalities available.

*(1) Using Gd-T1w scans only:* Survival analysis was employed using RaMP on the 3 data combinations on each tumor sub-compartment as well as on the tumor habitat. When employing $S_{t}^{S1,2}$ (training using Sites 1 and 2), significant differences were observed for $\begin{array}{lll} & I_{En\;}\left(\alpha =0,\;P<.0001\right),\;I_{Ed}\left(\alpha =0.54,\;P<.0001\right),\; & \;I_{NC}\left(\alpha =0,\;P<.0001\right),\;I_{H}\left(\alpha =0.02,\;P=.00037\right)\; \end{array}$. Top-performing features as selected by the regression models were then employed on $S_{v}^{S3}$, which yielded significant differences for $I_{Ed}\;\left(P=.04\right)$ and $I_{H}\;\left(P=.04\right).$ Similarly, when employing $S_{t}^{S1,3}$, significant differences were observed for $\begin{array}{lll} & I_{En\;}\left(\alpha =0.68,\;P<.0001\right),\;I_{Ed}\left(\alpha =0.84,\;P=.0005\right),\; & I_{NC}\left(\alpha =0,\;P<.0001\right),\;I_{H}(\alpha =0.04,\;P<.0001)\; \end{array}$. When the top-performing features were employed on $S_{v}^{S2}$, significant differences were observed for $I_{Ed}\;\left(P=.05\right)$, $I_{Enh}\;\left(P=.05\right)$, and $I_{H}\;\left(P=.03\right).$ Finally, when employing $S_{t}^{S2,3}$, significant differences were observed for $\begin{array}{lll} & I_{En\;}\left(\alpha =0.18,\;P<.0001\right),\;I_{Ed}\left(\alpha =0.5,\;P<.0001\right),\; & \;I_{NC}\left(\alpha =0,\;P<.0001\right),\;I_{H}\left(\alpha =0,\;P=.0025\right)\; \end{array}$. Top-performing features as selected by the regression models were then employed on $S_{v}^{S1}$, which yielded significant differences for $I_{En}\;\left(P=.03\right)$ and $I_{H}\;\left(P=.001\right).$ KM curves for all experiments that exhibited significant differences across the test sets are shown in Figure 2A.

*(2) Using the 3 MRI modalities*: When employing RaMP on $S_{t}^{S1,3}$, significant differences were observed for $\begin{array}{lll} & I_{Ed\;}\left(\alpha =0.02,\;P<.0001\right),\;I_{H}\left(\alpha =0.06,\;P<.0001\right),\; & \;I_{NC}\left(\alpha =0.54,\;P<.0001\right),\;\;I_{En}(\alpha =0.08,\;P<.0001)\; \end{array}$. When top-performing features were then employed on $S_{v}^{S2}$, significant differences were observed for $I_{Ed}\;\left(P=.026\right)$, $I_{NC}\;\left(P=.002\right)$, and $I_{H}\;\left(P=.026\right).$ Similarly, when employing RaMP on $S_{t}^{S2,3}$, significant differences were observed for $I_{En\;}\left(\alpha =0.2,\;P<.0001\right)\;and\;I_{NC}\left(\alpha =1,\;P=.001\right)$. Top-performing features as selected by the regression models were then employed on $S_{v}^{S1}$, which yielded significant differences for only $I_{NC}\;\left(P=.002\right).$ Finally, when employing RaMP on $S_{t}^{S1,2}$, significant differences were observed for $\begin{array}{lll} & I_{En\;}\left(\alpha =0,\;P=.001\right),\;I_{Ed}\left(\alpha =1,\;P<.0001\right),\; & I_{NC}\left(\alpha =0,\;P<.0001\right),\;I_{H}(\alpha =1,\;P=.001)\; \end{array}$. However, it is interesting to note that when the top features on $S_{v}^{S3}$ were employed, significant differences were not observed for any of the tumor regions. KM curves for all experiments that exhibited significant differences across the test sets are shown in Figure 2B.

**Figure 3.**
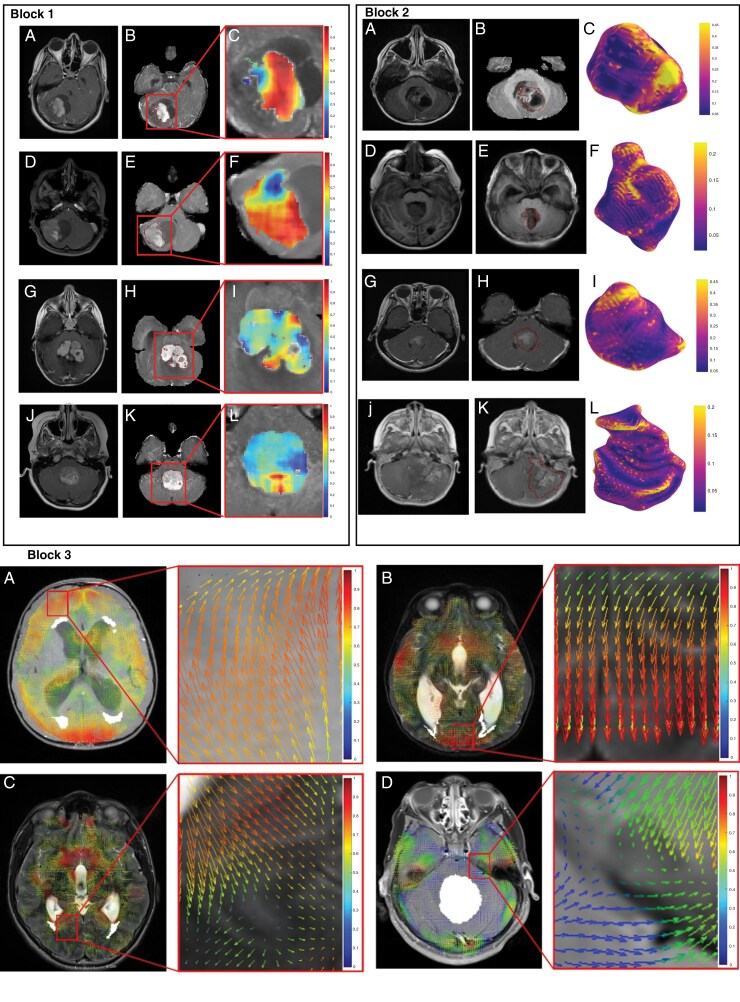
**Block 1:** MRI scans for high-risk (a, d) and low-risk (g, j) MB cases. Tumor borders for the scans are identified in b, e, h, and k. Heatmaps that illustrate hand-crafted textural features extracted for high-risk (c, f) and low-risk (i, l) MB cases. Entropy feature is shown in (c, i), whereas sum variance is shown in (f, l). **Block 2**: MRI scans for high-risk (a, d) and low-risk (g, j) MB cases. Tumor borders for the scans are identified in b, e, h, and k. Heatmaps that illustrate hand-crafted shape features extracted for high-risk (c, f) and low-risk (i, l) MB cases are shown. Total Curvature feature is shown in (c, i), whereas Curvedness feature is shown in (f, l). **Block 3:** Heatmaps that illustrate the deformation fields extracted for high-risk (a, b) and low-risk (c, d) MB cases. As shown in (a), (b), the heatmaps exhibit higher deformation values (in red) for high-risk cases, compared to the low-risk cases in (c), (d) that have lower deformation values (in blue, green).

The top features that were selected by the different penalized Cox models for the different data combinations are listed in Table 1Supplementary Document. Additionally, we report the performance metrics (*C*-index, HR, 95% CI) for each experiment that yielded significant differences on the test sets in Table 1Supplementary Document. Qualitative examples are shown in Figure 3 where heatmaps for high-risk and low-risk cases are illustrated for the top-performing features that constructed RaMP signature, across the different feature families (textural, morphology, deformations). Of note, there was no consistent top performer between using Gd-T1w images alone or all 3 sequences for the conducted survival analysis.

### Survival Risk Assessment Across Comparative Strategies

When employing $F_{S}$ alone for survival analysis (using Gd-T1 and all modalities), significant differences were observed only on the enhancing lesion of $S_{v}^{S3}\;$ and on the tumor habitat of $S_{v}^{S2}$ (Table 2 Supplementary Document). Similarly, employing $F_{\;}^{T}$ alone and $F_{\;}^{B}$ alone (using Gd-T1 and all modalities) resulted in significant differences across the 2 risk groups for some of the data combinations (Table 2 Supplementary Document). Of note, when employing Chang’s classification for survival analysis, significant differences were observed on both the training and test sets for one of the 3 data combinations ($S_{t}^{S2,3}\;\left(P=.02\right)$ and $S_{v}^{S1}\;\left(P=.0001\right)$). However, it is important to mention that modifications to treatment strategies for the patient cohorts have primarily been based on Chang’s classification. Additionally, survival risk assessment using molecular stratification alone did not yield any significant results across the test sites using the 3 data combinations. Similarly, combining Chang’s classification and molecular subgroup categorization for survival risk assessment did not yield significant differences across the different training and test sites. Interestingly, when we combined RaMP with clinical and molecular stratification, we did not observe significant differences across any of the data combinations. Such results were anticipated, since clinical and molecular information alone did not significantly contribute to survival risk assessment for our participating studies. Experiments across all the comparative strategies with results that exhibited significant differences across both risk groups are summarized in Table 2 Supplementary Document.

### Statistical Associations between RaMP and Clinical Risk-Stratification Approaches

When applying McNemar’s test to assess statistical associations between RaMP and Chang’s classification, a *P* value of .25 was obtained, indicating that no significant association exists between the 2 approaches. Similarly, applying Chi-square test for associations between RaMP and molecular subgroup classification yielded a *P* value of .3, indicating that no significant association exists between the 2 approaches.

## Discussion

This work presents a new RaMP for risk-stratification of pediatric MB patients on routine MR imaging. RaMP uniquely combines imaging features that quantify the intra- and peri-tumoral heterogeneity (using shape and textural features), as well as the structural deformations occurring on the healthy surrounding regions of the tumor (using biomechanical deformation magnitude and phase features). While the primary focus for survival risk assessment in MB has been molecular profiling, methylation, and whole chromosomal aberrations,^13,23,50^ a few traditional radiomic approaches have been employed so far to address this clinical problem.^24–34^ Our rigorous analysis showed statistical significance across patients from different institutions, while evaluating different cohort combinations for training and testing.

Notably, the best risk-stratification results were obtained when employing RaMP on Site 2 as a test set, with the 3 MRI sequences available (*n* = 83), where significant differences were obtained across edema, non-enhancing tumor + cystic core, and the tumor habitat on the test sets with *P* values of .02, .0035, .013 and *C*-indices of 0.7, 0.73, and 0.63, respectively.

Previous radiomic approaches that attempted to perform survival analysis for MB patients incorporated radiomic features as well as clinical and demographic variables within either machine learning classifiers or logistic regression models for risk assessment on smaller cohorts of studies.^24–34^ The recent study by Luo et al.^31^ employed radiomic features extracted from the PyRadiomics platform from 46 MB patients in a Support Vector Machine classifier, followed by combining the top selected features along with clinical variables into a multivariate logistic regression model for risk-stratification. This model yielded AUCs of 0.93 and 0.84 for training and held-out validation datasets, respectively. The study by Zhou et al.^32^ employed LASSO regression model with Random Survival Forest package for survival prediction of 217 MB patients. The top-performing model was the one that combined radiomic and clinical features (*C*-index = 0.75). Interestingly, our analysis using RaMP in combination with the clinical features (Chang’s and molecular stratification) did not yield statistically significant results for risk-stratification. This could be on account of the relatively smaller sample size of our study. This can also be on account of the non-uniformity of the treatment regimens for our retrospective studies. When comparing RaMP’s performance to that of the current risk assessment (Chang’s classification and molecular stratification), individually, no significant differences between risk groups were observed using molecular stratification, however significant differences were observed when Chang’s classification was applied on one of the data combinations [Table 2 Supplementary Document].

One would expect that our results would be better using the multi-parametric MRI sequences; however, we did not observe a consistent top performer between using Gd-T1w images alone or all 3 sequences for the analysis. This may be attributed to that the number of cases with the 3 sequences available was generally smaller across the 3 sites (*n* = 83 vs. 119 for Gd-T1w scans). We noticed that the *C*-indices were higher on the test sets when using the 3 sequences though.

Our top features that showed significant differences across the 2 risk groups were a combination of the 3 feature families [Table 2 supplementary document]. Notably, deformation angle values exhibited higher values for high-risk cases with significant differences between the two risk groups (eg *P* = .005 for mean of angles of deformation at 40 mm), suggesting the higher shift of voxels with respect to the tumor borders for aggressive tumors with poor survival. In addition, more highly curved surfaces (eg *P* = .04 for mean of sharpness) and higher entropy values were exhibited for high-risk cases with significant differences between the 2 risk groups (eg *P* = 0.05), suggesting the intra- and peri-tumoral heterogeneity of aggressive tumors with poor survival, compared to those with low risk of relapse.

Our approach had some limitations. First, while our cohort is multi-institutional, our sample size was still relatively small (*n* = 119). This limited the validation of RaMP, especially with having the 3 MRI sequences available for a subset of 83 patients. In addition, due to the retrospective nature of our studies, selection bias may have been introduced due to the lack of availability of all modalities for the MRI scans, in addition to missing parameters for a few cases, such as overall survival and the molecular subgroup information. Additionally, these retrospective cohorts did not receive uniform treatments, with treatment modifications often made based on Chang’s classification. This limitation made it challenging to assess the efficacy of Chang’s classification in risk-stratifying patients. Another known limitation with retrospective data is the non-homogeneity of the acquired MRI scans. However, we have rigorously conducted pre-processing to standardize the scan and minimize the inhomogeneities. To address the limitations associated with analyzing retrospective studies, we plan on curating subjects from clinical trials, where strict inclusion criteria are applied. This will guarantee that our cohorts are (1) uniformly treated; (2) having the same imaging modalities available and complete; (3) and having all clinical and molecular parameters available. The addition of more cases will also allow us to dedicate data from one site for independent validation (never seen by our trained models). Additionally, the clinical trial data will allow us to test the efficacy of our prognostic risk score in an individual patient setting and develop a personalized risk score that can help with guiding targeted therapies. We have already curated the MRI scans and clinical parameters for one clinical trial for MB therapy de-escalation and on our way to curate the studies from a clinical trial for therapy intensification.

To summarize, this work presented a radiomic prognostic score for accurate risk-stratification in pediatric medulloblastoma. Our results showed that radiomic approaches may offer valuable prognostic insights toward reliable risk-stratification, that may be overlooked by current clinical risk-stratification or molecular characteristics.

## Supplementary Material

vdaf107_suppl_Supplementary_Materials

## Data Availability

The MRI scans obtained from Sites 1 and 2 are protected through institutional compliance at the local institutions. The clinical repository of these patient scans can be shared per specific IRB requirements. Upon reasonable request, a data sharing agreement can be initiated between the interested parties and the clinical institution following institution-specific guidelines. Data from Site 3 was obtained from Children’s Brain Tumor Network (CBTN), based on an established agreement between the authors and CBTN. CBTN membership can be obtained following the guidelines provided on their website to obtain access to the scans as well.
