# Supplementary material for: Radiomic-based prognostic score for survival risk-stratification in pediatric medulloblastoma tumors: A multi-institutional study
Source: Neurooncol Adv. 2025 Jun 5;7(1):vdaf107. doi: 10.1093/noajnl/vdaf107 (PMC12284643; doi:10.1093/noajnl/vdaf107)
Supplement: vdaf107_suppl_Supplementary_Materials [file vdaf107_suppl_supplementary_materials.docx]

| **Table 1:** performance metrics (C-Index, HR, 95% CI) for each survival analysis experiment that employed the RaMP score and yielded significant differences on the test sets | | | | | |
| --- | --- | --- | --- | --- | --- |
|  | Performance metrics | | | |  |
| Site for testing | C-Index | HR | 95% CI | P | Top performing features per tumor sub-compartment |
| $S_{v}^{S3}$, Gd-T1w | 0.62 | 0.47 | 0.10 – 0.54 | 0.04 | $\mathcal{F}_{Ed}^{S}$: mean (sharpness), kurtosis (shape index), Orientation,  ${\mathcal{F}_{Mag}^{B}}$: standard deviation (60 mm), Angle Bin 3: 45, 50, 55 mm, ${\mathcal{F}_{Ed}^{T}}$: skewness (Laws), median (Laws). |
|  | 0.65 | 0.48 | 0.20 – 1.0 | 0.04 | $\mathcal{F}_{Mag}^{B}$: standard deviation (60 mm), ${\mathcal{F}_{\theta}^{B}:}$ kurtosis (angle 60 mm), Angle Bin 1 (50, 55 mm), Angle Bin 4 (20 – 55 mm). |
| $S_{v}^{S2}$, Gd-T1w | 0.60 | 0.50 | 0.10 – 0.40 | 0.05 | $\mathcal{F}_{Mag}^{B}$: skewness (angle 60 mm), $\mathcal{F}_{\theta}^{B}:$kurtosis (angle 60 mm) Angle Bin 1 (50, 55 mm), Angle Bin 3 (40 mm). |
|  | 0.59 | 0.50 | 0.10 – 0.40 | 0.05 | $\mathcal{F}_{Mag}^{B}$**:** median (magnitude 60 mm), skewness (magnitude 60 mm), $\mathcal{F}_{\theta}^{B}:$ kurtosis (angle 60 mm), $\mathcal{F}_{En}^{T}$**:** kurtosis (Gabor), mean (Laws), skewness (Laws). |
|  | 0.62 | 0.49 | 0.13 – 0.60 | 0.03 | ${\mathcal{F}_{H}^{S}}$**:** Eccentricity, Elongation, $\mathcal{F}_{Mag}^{B}$: variance, skewness, kurtosis (magnitude 35 mm), $\mathcal{F}_{\theta}^{B}:$kurtosis (angle 60 mm), Angle Bin 1 (15, 20, 25, 30 mm), ${\mathcal{F}_{H}^{T}}$: skewness (Gradient), skewness (Haralick), skewness (Gabor), median, skewness, variance (Laws). |
| $S_{v}^{S1}$, Gd-T1w | 0.60 | 0.50 | 0.20 – 0.80 | 0.04 | $\mathcal{F}_{\theta}^{B}$**:** Angle Bin 1 (15 mm), Angle Bin 2 (5, 55, 60 mm), Angle Bin 4 (30 - 60 mm), $\mathcal{F}_{En}^{T}$**:** mean (Haralick), skewness (Gabor), skewness (Laws). |
|  | 0.70 | 0.40 | 0.20 – 1.0 | <0.01 | $\mathcal{F}_{H}^{S}$**:** Eccentricity, $\mathcal{F}_{Mag}^{B}$: kurtosis (magnitude 35 mm), $\mathcal{F}_{\theta}^{B}:$ kurtosis (angle 60 mm), Angle Bin 1 (15,30 mm), ${\mathcal{F}_{H}^{T}}$: skewness (Gradient), skewness (Haralick), skewness, kurtosis (Gabor), kurtosis (Laws). |
| $S_{v}^{S2}$, Gd-T1w, T2w, FLAIR | 0.70 | 0.34 | 0.10 – 1.20 | 0.03 | ${\mathcal{F}_{Mag}^{B}:}$variance, skewness (magnitude 30 mm), variance (magnitude 40 mm), $\mathcal{F}_{\theta}^{B}:$Angle Bin 2 (10,15 mm), Angle Bin 3 (45,50 mm), ${\mathcal{F}_{Ed}^{T}}$: **T1:** variance, kurtosis (Gradient), median (Haralick), mean, variance (Gabor), kurtosis (Laws), **FLAIR**: Kurtosis (Laws). |
|  | 0.73 | 0.34 | 0.10 – 1.20 | 0.03 | $\mathcal{F}_{Mag}^{B}$: **T1**: mean (magnitude 30 mm), $\mathcal{F}_{\theta}^{B}:$Angle Bin 1 (50, 55 mm), **T2** : $\mathcal{F}_{Mag}^{B}:$mean (magnitude 30, 35 mm), $\mathcal{F}_{\theta}^{B}:$median (Angle 45 mm), skewness (Angle 45 mm), FLAIR; $\mathcal{F}_{Mag}^{B}:$mean (magnitude 30 mm), $\mathcal{F}_{H}^{T}$**:** **T1:** kurtosis, median (Laws), T2: variance (Laws), **FLAIR**: variance (Laws), kurtosis (Collage : difference variance). |
|  | 0.63 | 0.34 | 0.10 – 1.0 | 0.03 | $\mathcal{F}_{Mag}^{B}$: **T1**: mean (magnitude 60 mm), $\mathcal{F}_{\theta}^{B}:$Angle Bin 2 (10, 15 mm), mean (magnitude 35 mm), **T2**: Angle Bin 1(45, 50, 55 mm), **FLAIR**: $\mathcal{F}_{Mag}^{B}:$median (magnitude 35 mm), median (magnitude 60 mm), ${\mathcal{F}_{NC}^{T}}:$**T2** : skewness (Laws), **FLAIR**: mean, kurtosis (Laws). |
| $S_{v}^{S1}$, Gd-T1w, T2w, FLAIR | 0.64 | 0.38 | 0.10 – 0.80 | <0.01 | ${\mathcal{F}_{NC}^{S}}$: Eccentricity, Elongation, Compactness, $\mathcal{F}_{Mag}^{B}$: **T1**: mean (magnitude 30 mm), $\mathcal{F}_{\theta}^{B}:$Angle Bins 1, 2,3 (10, 15, 20 mm), **T2**: Angle Bin 1(15, 30 mm), **FLAIR**: median (magnitude 40, 45 mm), median, kurtosis (magnitude 60 mm), $\mathcal{F}_{NC}^{T}$: **T1**: Skewness, median (Laws), median (Gabor), **T2**: skewness (Laws), **FLAIR**: mean, kurtosis (Laws), kurtosis (Gabor), Kurtosis (Gradient). |

| **Table 2:** performance metrics (C-Index, HR, 95% CI) for each survival analysis experiment that employed the individual feature families and yielded significant differences on the test sets. | | | | | | | | |
| --- | --- | --- | --- | --- | --- | --- | --- | --- |
| Feature Family | Performance Metrics | | | | | | | |
|  | Gd-T1w | | | Gd-T1w, T2w, FLAIR | | | | |
|  | Site for testing | Tumor region | Performance metrics | Site | Tumor region | | Performance metrics | |
| $\mathcal{F}^{S}$ | $S_{v}^{S3}$  $S_{v}^{S2}$ | $I_{En}$  $I_{H}$ | p = 0.007, C-Index = 0.65, HR = 0.38, CI = 0.24 – 0.69  p = 0.02, C-Index = 0.59, HR = 0.49, CI = 0.20 – 0.86 | $S_{v}^{S3}$  $S_{v}^{S3}$ | | $I_{En}$  $I_{H}$ | | p = 0.007, C-Index = 0.65, HR = 0.38, CI = 0.24 – 0.69  p = 0.02, C-Index = 0.59, HR = 0.49, CI = 0.20 – 0.86 |
| $\mathcal{F}^{T}$ | $S_{v}^{S2}$  $S_{v}^{S1}$ | $I_{En}$  $I_{Ed}$ | p = 0.0065, C-Index = 0.66, HR = 0.40, CI = 0.08 – 0.40  p = 0.00096, C-Index = 0.64, HR = 0.37, CI = 0.10 – 0.48 | $S_{v}^{S3}$  $S_{v}^{S2}$  $S_{v}^{Site1}$ | | $I_{H}$  $I_{En}$  $I_{Ed}$  $I_{En}$  $I_{H}$  $I_{En}$ | | p = 0.05, C-Index = 0.55, HR = 0.50, CI = 0.18 – 0.94  p = 0.044, C-Index = 0.56, HR = 0.46, CI = 0.10 – 0.50  p = 0.043, C-Index = 0.70, HR = 0.37, CI = 0.05 – 0.68  p = 0.038, C-Index = 0.78, HR = 0.35, CI = 0.12 – 1.40  p = 0.0067, C-Index = 0.65, HR = 0.41, CI = 0.15 – 0.71  p = 0.0068, C-Index = 0.65, HR = 0.40, CI = 0.08 – 0.40 |
| $\mathcal{F}_{Mag}^{B}$ | $S_{v}^{S2}$  $S_{v}^{S1}$ | $I_{B}$  $I_{B}$ | p = 0.026, C-Index = 0.67, HR = 0.48, CI = 0.18 – 0.8  p = 0.05, C-Index = 0.62, HR = 0.54, CI = 0.28 – 1.13 | $S_{v}^{S2}$  $S_{v}^{S1}$ | | $I_{B}$  $I_{B}$ | | p = 0.01, C-Index = 0.69, HR = 0.30, CI = 0.10 – 1.6  p = 0.03, C-Index = 0.64, HR = 0.50, CI = 0.10 – 0.40 |
| Chang’s classification | $S_{v}^{S1}$ |  | p = 0.0001  C-Index = 0.73  HR = 0.54  CI = 0.45 – 2.0 | N/A | | | | |
